# Association between maternal vitamin D supplementation during pregnancy and the risk of acute respiratory infections in offspring: a systematic review and meta-analysis

**DOI:** 10.1016/j.eclinm.2025.103682

**Published:** 2025-12-04

**Authors:** David A. Jolliffe, Nicklas Brustad, Bo Chawes, Cyrus Cooper, Stefania D'angelo, Nicholas C. Harvey, Augusto A. Litonjua, Rebecca Moon, Shaun K. Morris, John D. Sluyter, Scott T. Weiss, Adrian R. Martineau

**Affiliations:** a Blizard Institute, Faculty of Medicine and Dentistry, Queen Mary University of London, London, UK; b COPSAC, Copenhagen Prospective Studies on Asthma in Childhood, Herlev and Gentofte Hospital, University of Copenhagen, Copenhagen, Denmark; c MRC Lifecourse Epidemiology Unit, University of Southampton, Southampton, UK; d Department of Pediatrics Golisano Children's Hospital, Pediatric Pulmonary Division, University of Rochester Medical School, Rochester, NY, USA; e Division of Infectious Diseases, Child Health Evaluative Sciences & Centre for Global Child Health, Hospital for Sick Children, 686 Bay Street, Toronto, ON, Canada; f School of Population Health, University of Auckland, Auckland, New Zealand; g Department of Medicine, Channing Division of Network Medicine, Brigham and Women's Hospital, Harvard Medical School, Boston, MA, USA; h NIHR Southampton Biomedical Research Centre, University of Southampton and University Hospital NHS Foundation Trust, Southampton, UK

**Keywords:** Maternal vitamin D supplementation, Acute respiratory infections, Pregnancy, Offspring

## Abstract

**Background:**

Acute respiratory infections (ARIs) are a leading cause of mortality in infants. Vitamin D supports innate antimicrobial effector mechanisms in leucocytes and respiratory epithelium. Maternal vitamin D supplementation during pregnancy has been proposed as a preventive strategy, however, an up-to-date synthesis of available data from randomised controlled trials (RCTs) has not been conducted.

**Methods:**

We conducted a systematic review and meta-analysis of aggregate data from RCTs of maternal vitamin D supplementation for prevention of ARIs in offspring. Data were analysed using a random-effects model. We searched MEDLINE, EMBASE, the Cochrane Central Register of Controlled Trials, Web of Science and the ClinicalTrials.gov from database inception to 5th August 2025. No language restrictions were imposed. Double-blind RCTs of maternal vitamin D supplementation, with placebo or lower-dose vitamin D control, were eligible if approved by Research Ethics Committee and if ARI incidence in offspring was collected prospectively and pre-specified as an efficacy outcome. Sub-group analyses were done to determine whether effects of maternal vitamin D supplementation on offspring ARI risk varied according to maternal baseline circulating 25-hydroxyvitamin D (25 [OH]D) concentrations (<25 nmol/L, 25–49.9 nmol/L, 50–74.9 nmol/L, or ≥75 nmol/L). The study was registered with PROSPERO, CRD42024527191.

**Findings:**

Our search identified 405 unique studies, of which 4 RCTs (3678 participants) were eligible and included. For the primary comparison of any maternal vitamin D supplementation vs. placebo, the intervention did not significantly affect overall ARI risk in offspring (incidence rate ratio [IRR] 1.01, 95% CI 0.98–1.03, P = 0.66; 4 studies; I^2^ 14.5%, absolute effects from GRADE assessment: 0.05 higher rate in vitamin D arm; moderate quality finding). Pre-specified subgroup analysis did not reveal evidence of effect modification by maternal baseline vitamin D status: <25 nmol/L group: IRR 1.12, 95% CI 0.98–1.27 (607 participants in 4 studies, I^2^ 47.8%) vs. 25–49.9 nmol/L group: IRR 1.04, 95% CI 0.96–1.13 (1154 participants in 4 studies, I^2^ 68.5%) vs. 50–74.9 nmol/L group: IRR 1.00, 95% CI 0.93–1.08 (789 participants in 4 studies, I^2^ 64.9%) vs. ≥75 nmol/L group: IRR 0.97, 95% CI 0.89–1.06 (505 participants in 4 studies, I^2^ 47.6%). A funnel plot did not indicate the presence of publication bias or small-study effects (P = 0.71, Egger's test).

**Interpretation:**

Our analysis of current data does not support routine antenatal vitamin D supplementation for the prevention of ARI in offspring. Key limitations of the study were the administration of a low dose vitamin D standard-of-care in some populations which may have attenuated effects of the intervention, and heterogeneity in ARI case definitions which may have introduced misclassification bias. Targeted supplementation in deficient populations may warrant further investigation.

**Funding:**

None.

**Research in context**

**Evidence before this study**

We identified two relevant meta-analyses of observational birth cohort studies through informal consultation of the literature (including MEDLINE, EMBASE, and Cochrane Database of Systematic Reviews, with no language restrictions, from inception up to Dec 31st, 2024), using general search terms: ‘*vitamin D’, ‘supplementation’, ‘maternal/pregnancy’, ‘respiratory infection*’. One meta-analysis of 9 cohorts reported no significant association between highest versus lowest maternal 25-hydroxyvitamin D [25(OH)D] categories and offspring ARI risk (moderate heterogeneity; no evidence of publication bias). A second, more recent meta-analysis of 13 cohorts reported a significant inverse association but with high heterogeneity and evidence of publication bias. Prior to this study, no meta-analysis of RCTs had evaluated the effects of maternal vitamin D supplementation exclusively in mothers supplemented during pregnancy on risk of ARI in offspring who did not receive supplementation beyond the standard of care, either overall or stratified by maternal baseline vitamin D status. Given the inconsistency of observational findings and the absence of RCT-based meta-analysis—particularly one examining effect modification by baseline vitamin D status—we undertook this study to address this evidence gap.

**Added value of this study**

Our meta-analysis of aggregate data from 3678 participants in 4 randomised controlled trials, stratified by baseline 25(OH)D concentration, suggest there is no effect of maternal vitamin D supplementation against offspring acute respiratory infection risk overall, or in sub-groups defined by baseline maternal vitamin D status.

**Implications of all the available evidence**

Meta-analysis including the latest available RCT data does not support routine antenatal vitamin D supplementation for the prevention of ARI in offspring. Key limitations of the study were the administration of a low dose vitamin D standard-of-care in some populations which may have attenuated effects of the intervention, and heterogeneity in ARI case definitions which may have introduced misclassification bias. Future research which avoids these limitations and targets more deficient populations is warranted.

## Introduction

Acute respiratory infections (ARIs) are typically defined as any infection of the respiratory tract with symptom duration up to 21 days. Their impact is particularly pronounced in paediatric populations: the 2020 Global Burden of Disease study reported infectious diseases to be the leading cause of mortality in children aged under 5^1^ and lower respiratory infections (LRI) have been ranked the highest category of infection-related mortality in this age group.^2^

Findings from laboratory studies suggest that vitamin D may reduce ARI risk by supporting innate immune responses.^3^ In the context of maternal supplementation, protection could be extended to offspring in the neonatal period, during which serum 25-hydroxyvitamin D (25 [OH]D) concentrations reflect maternal levels.^4^ Beyond the neonatal period, epigenetic mechanisms induced by maternal supplementation, such as DNA methylation, could impact long-term offspring immune function.^5^ Moreover, the role of the epigenome on immune responses to vaccines is a rapidly growing area of interest.^6^ Gestational vitamin D supplementation might impact the protective efficacy of childhood vaccinations, indirectly influencing risk of ARI in offspring.

Findings from observational studies investigating associations between maternal vitamin D status and risk of ARI in offspring are mixed. One meta-analysis of 9 birth cohort studies reported no significant association between highest vs. lowest categories of maternal 25(OH)D concentrations and risk of ARI in offspring, with moderate heterogeneity (*I*^*2*^ = 66%) and no evidence of publication bias (Egger's test, P = 0.49).^7^ In contrast, a more recent meta-analysis of 13 birth cohort studies reported a significant inverse association between maternal 25(OH)D concentrations and risk of ARI in offspring, albeit with greater heterogeneity across synthesised studies (*I*^*2*^ = 83%) and significant evidence of publication bias (Egger's test, P = 0.005).^8^

Data from randomised controlled trials (RCTs) could resolve inconsistencies in observational findings, however they are also conflicting and have not been synthesised in a meta-analysis investigating overall and subgroup effects. We therefore sought data from these studies for inclusion in a meta-analysis of stratified aggregate data (trial-level, stratified by baseline vitamin D status). Our objectives were to determine whether maternal vitamin D supplementation during pregnancy reduced overall risk of ARI in offspring, and to evaluate whether effects of vitamin D on risk of ARI in offspring varied according to maternal baseline 25(OH)D concentration at enrolment.

## Methods

### Search strategy and selection criteria

This systematic review and meta-analysis was conducted in accordance with PRISMA 2020 guidelines. Two investigators (ARM and DAJ) searched MEDLINE, EMBASE, the Cochrane Central Register of Controlled Trials (CENTRAL), Web of Science and the ClinicalTrials.gov registry using the electronic search strategies described in the Appendix (Section 1, p3-5). Searches included studies registered from database inception to 5th August 2025. No language restrictions were imposed. These searches were supplemented by searching review articles and reference lists of trial publications. Collaborators were asked if they knew of any additional eligible RCTs.

Double-blind randomised controlled trials of maternal supplementation with vitamin D_3_, vitamin D_2_ or 25(OH)D of any duration during pregnancy, with a placebo or blinded lower-dose vitamin D control for the primary prevention of ARI in offspring, were eligible for inclusion if they had been approved by a Research Ethics Committee and if data on incidence of ARI were collected prospectively and pre-specified as an efficacy outcome. The latter requirement was imposed to minimise misclassification bias (prospective capture of ARI was deemed more likely to be sensitive and specific for this outcome).

Methods were pre-specified in a protocol that was registered with the PROSPERO International Prospective Register of Systematic Reviews (registration no. CRD42024527191) (click here). Details of Research Ethics Committee approvals to conduct the primary trials included in this study are included in Appendix (Section 4, p8). One trial (MAVIDOS) contributed previously unpublished, prospectively collected data to the meta-analysis; the trial was approved by the Southampton and South West Hampshire Research Ethics Committee (ISRCTN:82927713; EUDRACT:2007-001716-23) and all participants provided written informed consent.

### Data analysis

Details of the data extraction process can be found in Appendix (Section 2, p6). Two investigators (ARM and DAJ) searched articles independently and reviewed extracted data. Any discordance was resolved through discussion. A third investigator (JDS) verified extracted data. The primary outcome of the meta-analysis was the rate of ARI in offspring. The definition of ARI encompassed events classified as upper respiratory infection (URI), lower respiratory infection (LRI) and ARI of unclassified location (i.e. infection of the upper and/or lower respiratory tract). Secondary outcomes were the incidence of URI and LRI, analysed separately. Pre-specified data on the incidence of acute respiratory infections was collected but not previously published for one trial.^9^ The additional secondary outcomes requested (Appendix Section 2, p6) were not sufficiently available to be meta-analysed.

Data were analysed by DAJ; results were checked and verified by JDS. Our meta-analysis approach followed published guidelines.^10^ The primary comparison was of participants randomised to any vitamin D supplement vs. placebo: this was performed for all of the outcomes listed above. For one trial that included high-dose, medium-dose, low-dose and placebo arms,^11^ data from intervention arms were pooled. The log incidence rate ratio and its standard error were calculated for each outcome within each trial from the total number of events and time at risk, for participants in the intervention vs. control arm. This was done in accordance with the Cochrane Handbook's guidelines.^12^ Log incidence rate ratios were then meta-analysed in a random-effects model using the Metan package^13^ within STATA IC v14.2 to obtain an overall incidence rate ratio with a 95% confidence interval (CI) and a measure of heterogeneity summarised by the I^2^ statistic and its associated P value.

We used the Cochrane Collaboration Risk of Bias tool^14^ to assess the following variables: sequence generation, allocation concealment, blinding of participants, personnel and outcome assessors, completeness of outcome data, evidence of selective outcome reporting, and other potential threats to validity. Study quality was assessed independently by two investigators (ARM and DAJ). Any discrepancies were resolved by discussion. For the primary analysis, the likelihood of publication bias was investigated through the construction of a contour-enhanced funnel plot.^15^ We used the five Grading of Recommendations, Assessment, Development and Evaluation (GRADE) considerations (study limitations, consistency of effect, imprecision, indirectness and publication bias)^16^ to assess the quality of the body of evidence contributing to analyses. This applied to both the primary efficacy outcome and secondary outcomes of our meta-analysis. We conducted a sensitivity analysis for the primary outcome, excluding one RCT where risk of bias was assessed as being unclear.^9^

To explore reasons for heterogeneity of effect of the intervention between trials we performed a stratified analysis according to maternal baseline vitamin D status (serum 25 [OH]D < 25 *vs.* 25–49.9 *vs.* 50–74.9 *vs.* ≥75 nmol/L). The thresholds for baseline 25(OH)D concentration were selected *a priori* on the basis that they represent cut-offs that are commonly used to distinguish profound vitamin D deficiency (<25 nmol/L), moderate vitamin D deficiency (25–49.9 nmol/L) and potentially sub-optimal vitamin D status (50–74.9 nmol/L).^17^

### Role of funding source

This study was conducted without external funding.

All authors had access to the study data. DAJ and ARM had final responsibility for the decision to submit the work for publication.

## Results

### Study selection and data obtained

**Fig. 1:**
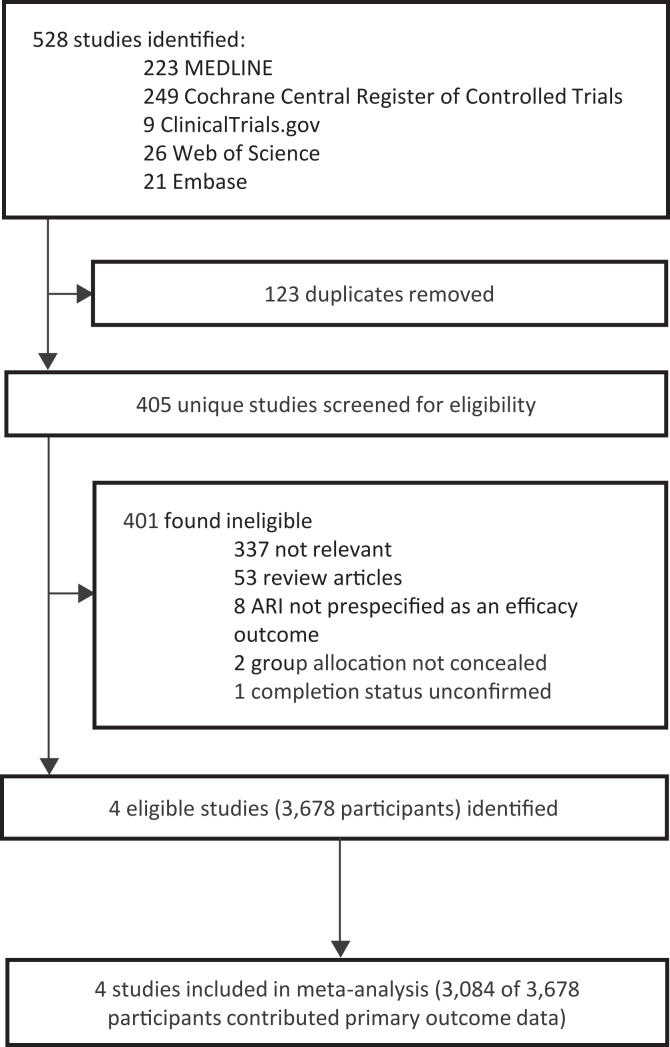
Flow chart of study selection.

The study selection process is illustrated in Fig. 1. Our search (studies published from inception to 5th August 2025) identified a total of 405 unique studies that were assessed for eligibility, of which 4 studies^9^^,^^11^^,^^18^^,^^19^ with a total of 3678 randomised participants fulfilled eligibility criteria. All four of the eligible studies identified compared effects of a vitamin D regimen vs. placebo. Data for the primary outcome (incidence rate of ARI in offspring) were obtained for 3084/3678 (83.8%) participants in the 4 studies.

### Study and participant characteristics

Characteristics of the 4 studies contributing data to this meta-analysis are presented in Table 1. Trials were conducted in 4 different countries on 3 continents, and enrolled pregnant women with a gestational age of 10–24 weeks. 1582/3291 (48.1%) of offspring were female. Baseline serum 25(OH)D concentrations were determined in all trials: mean maternal baseline 25(OH)D concentrations for different trials ranged from 27.7 to 77.4 nmol/L (to convert to ng/mL, divide by 2.496). All studies administered oral vitamin D_3_ to pregnant women allocated to intervention arms; 3 studies administered this as daily doses, whilst one study administered it as a weekly dose. Three of the four trials were conducted in countries with a maternal standard of care of up to 10 μg vitamin D_3_ daily. Trial duration ranged from 6.5 months to 7 years. Incidence of ARI was the primary outcome for 1 study, and a secondary outcome for 3 studies.

**Table 1:** Characteristics of trials and their participants.

Characteristic	Study first author, year			
	Chawes, 2016^18^	Cooper, 2016^9^	Litonjua, 2016^19^	Morris, 2021^11^
Setting	Denmark	UK	USA	Bangladesh
Participants	Mother-offspring pairs	Mother-offspring pairs	Mother-offspring pairs	Mother-offspring pairs
Mean Maternal age at enrolment, years (s.d.) [range]	31.8 (4.3) [18.5–47.8]	30.5 (5.2) [18.6–44.8]	27.3 (5.5) [18.0–39.5]	23.2 (4.2) [18.0–40.0]
Offspring Male: Female	298:283	517:448	420:386	474:465
25 (OH)D assay, EQA scheme	LC-MS/MS, DEQAS	RIA (Diasorin), DEQAS	CLA (DiaSorin)/LC-MS/MS	LC-MS/MS, DEQAS
Maternal baseline 25(OH)D sampling timepoint, weeks of gestation	24	14–17	10–18	17–24
Mean Maternal baseline 25(OH)D at enrolment, nmol/L (s.d.)	77.4 (25.0)	46.3 (17.4)	57.4 (25.5)	27.6 (14.3)
Maternal 25(OH)D < 25 nmol/L at enrolment (%)	8/581 (1.4)	118/1134 (10.4)	–	449/935 (48.0)
Mean Maternal attained 25(OH)D (at birth), intervention arm, nmo/L (s.d.)	107.3 (34.9)	–	–	93.8 (29.2) ^(a)^
Mothers randomised, Intervention: Control (total)	315:308 (623)	565:569 (1134)	442:439 (881)	780^(b)^: 260 (1040)
Oral dose of vitamin D_3_, intervention arm	60 μg daily + standard care up to 10 μg daily	25 μg + standard care up to 10 μg daily	100 μg daily + standard care up to 10 μg daily	0.41 mg weekly^(c)^
Control	Placebo + standard care up to 10 μg daily	Placebo + standard care up to 10 μg daily	Placebo + standard care up to 10 μg daily	Placebo
Trial follow-up duration	3 yrs	7 yrs	3 yrs	6.5 mo
ARI definition	Physician confirmed ARI	ARI: parent-reported	ARI: parental-report of physician's diagnosis	ARI: lab confirmed
ARI primary or secondary outcome?	Secondary	Secondary	Secondary	Primary
N Offspring contributing data/N Mothers randomised (%)	572/623 (93.3)	767/1134 (67.6)	806/881 (91.5)	939/1040 (90.3)

(a) Pooled mean and standard deviation for the low/med/high dose intervention groups; (b) Total number of participants in the low/med/high dose intervention groups; (c) Mean dose for the high (0.70 mg), medium (0.42 mg) and low (0.11 mg) dose intervention groups.

Abbreviations: DEQAS, vitamin D external quality assessment scheme; LC-MS/MS, liquid chromatography tandem mass spectrometry; RIA, radioimmunoassay; CIA, chemiluminescence assay.

### Risk of bias within studies

Details of the risk of bias assessment are provided in Appendix Table S2, p10. One trial^9^ was assessed as being at unclear risk of bias due to high loss to follow-up (367/1134 [32.4%] of randomised participants did not complete all symptom questionnaires). The remaining 3 trials were assessed as being at low risk of bias for all seven aspects assessed.

### Overall results, primary outcome

**Fig. 2:**
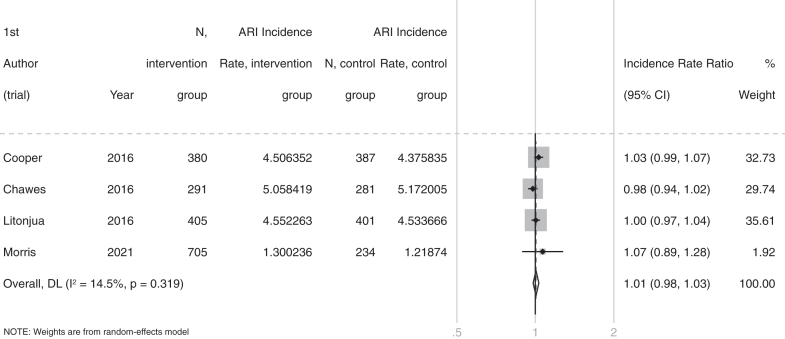
Forest plot of placebo-controlled RCTs of maternal vitamin D supplementation during pregnancy whose offspring experience respiratory infections: rate analysis.

For the primary comparison of any vitamin D supplement vs. placebo control, supplementation did not result in a statistically significant reduction in the rate of offspring experiencing ARI (incidence rate ratio [IRR] 1.01, 95% CI 0.98–1.03, P = 0.66; 3084 participants in 4 studies; Fig. 2, Table 2). Between-trial heterogeneity was low: I^2^ = 14.5% (P-value for heterogeneity: 0.32). GRADE analysis (Appendix Table S3) estimated the anticipated absolute rate of infections per person-year to be 0.05 higher in the vitamin D arm (−0.09 lower to 0.18 higher), compared to the placebo arm. This finding was estimated to be of moderate quality.

**Table 2:** Rate of incident acute respiratory infections among offspring of mothers randomised to receive vitamin D vs. placebo during pregnancy, by allocation: overall and by baseline maternal circulating 25-hydroxyvitamin D concentration.

	No. trials	Intervention group			Control group			Incidence rate ratio (95% CI)	I^2^%	P for heterog-eneity
		Offspring, N	Total ARI events	Person-years	Offspring, N	Total ARI events	Person-years			
**Overall**	4	1781	15,408	3570.7	1303	14,879	3294.7	1.01 (0.98–1.03)	14.5	0.32
**Maternal baseline 25(OH)D, nmol/L**										
<25	4	417	1361	413.6	190	1059	294.9	1.12 (0.98–1.27)	47.8	0.12
25–49.9	4	675	5211	1233.0	479	4978	1146.3	1.04 (0.96–1.13)	68.5	0.023
50–74.9	4	409	5030	1106.1	380	5092	1100.9	1.00 (0.93–1.08)	64.9	0.036
≥75	4	263	3683	775.4	242	3613	719.8	0.97 (0.89–1.06)	47.6	0.13

Abbreviations: CI, confidence interval; N, number; ARI, acute respiratory infection; 25(OH)D, 25-hydroxyvitamin D; nmol/L, nanomoles/litre.

### Sub-group analysis, primary outcome

To investigate reasons for the observed heterogeneity of effect for the primary comparison of any vitamin D supplement vs. placebo control, we stratified this analysis by maternal baseline vitamin D status. Results are presented in Table 2 and Appendix Fig. S2, p13. No statistically significant effect of vitamin D was seen for participants with baseline 25(OH)D < 25 nmol/L (IRR 1.12, 95% CI 0.98–1.27; 607 participants in 4 studies, I^2^ 47.8%), 25–49.9 nmol/L (IRR 1.04, 95% CI 0.96–1.13; 1154 participants in 4 studies, I^2^ 68.5%), 50–74.9 nmol/L (IRR 1.00, 95% CI 0.93–1.08; 789 participants in 4 studies, I^2^ 64.9%), or ≥75 nmol/L (IRR 0.97, 95% CI 0.89–1.06; 505 participants in 4 studies, I^2^ 47.6%).

### Secondary outcomes

Results of secondary outcomes are presented in Table 3 and illustrated in Appendix Fig. S3, p14 and Fig. S4, p15. Overall, vitamin D supplementation did not have a statistically significant effect on the rates of URI or LRI.

**Table 3:** Rate of incident upper and lower respiratory infections among offspring of mothers randomised to receive vitamin D vs. placebo during pregnancy, by allocation.

	No. trials	Intervention group			Control group			Incidence rate ratio (95% CI)	I^2^%	P for heterog-eneity
		Offspring, N	Total ARI events	Person-years	Offspring, N	Total ARI events	Person-years			
Upper respiratory infections	4	1781	14,578	3570.7	1303	13,993	3294.7	1.01 (0.98–1.04)	35.0	0.20
Lower respiratory infections	4	1781	901	3570.7	1303	908	3294.7	0.95 (0.82–1.09)	47.6	0.13

Abbreviations: CI, confidence interval; N, number; ARI, acute respiratory infection.

### Risk of bias across studies

A funnel plot for the incidence rate ratios of participants experiencing ARI (Appendix Fig. S5, p16) does not show significant asymmetry that would suggest publication bias or small-study effects. This was confirmed with an Egger's regression test^20^ (P = 0.71).

### Sensitivity analysis

Results of a sensitivity analysis excluding one study assessed as being of unclear risk of bias are presented in Appendix Table S4, p12. It did not reveal a statistically significant protective effect of vitamin D supplementation (IRR 0.99, 95% CI 0.97–1.02; 2317 participants in 3 studies). Leave-one-out analysis confirmed the robustness of null results (p > 0.05 in all models, Appendix Figure S6).

## Discussion

To our knowledge, this systematic review and meta-analysis represents the most comprehensive synthesis to date of RCT data evaluating the effect of maternal vitamin D supplementation during pregnancy on the risk of ARIs in offspring. By aggregating stratified data from 3678 participants across four RCTs, we found no significant reduction in overall ARI incidence among offspring of mothers receiving vitamin D supplementation compared to placebo (IRR 1.01, 95% CI 0.98–1.03, absolute effects: 0.05 higher rate of infection in vitamin D arm). Subgroup analyses stratified by maternal baseline 25-hydroxyvitamin D (25 [OH]D) concentrations similarly revealed no evidence of effect modification. Including in populations with profound vitamin D deficiency (<25 nmol/L). These null findings persisted across sensitivity analyses and secondary outcomes (URI, LRI), underscoring the robustness of the results.

Our results contrast with observational studies reporting inverse associations between maternal 25(OH)D concentrations and offspring ARI risk.^7^^,^^8^ This discrepancy may reflect residual or unmeasured confounding in observational designs, due to socioeconomic factors, diet, or genetic variation, which RCTs inherently mitigate. Alternatively, methodological limitations in the included trials could obscure a true biological effect. For instance, standard-of-care vitamin D supplementation (up to 10 μg/day) provided to both intervention and control arms in three trials may have attenuated inter-arm differences in maternal 25(OH)D concentrations at follow-up, particularly in populations with moderate baseline deficiency. Additionally, adherence issues, non-daily dosing or failure to elevate maternal 25(OH)D concentrations into a therapeutic range (e.g., >75 nmol/L) might have limited effects of the interventions studied. Biological plausibility for a protective effect exists, as mechanistic studies suggest vitamin D modulates neonatal innate immunity and epigenetic programming.^3^^,^^5^ However, the absence of clinical benefit in this meta-analysis implies that such mechanisms, if operative, may not translate to measurable reductions in ARI risk under real-world trial conditions.

Strengths of this work include the inclusion of data from all eligible RCTs, stratified analyses by maternal baseline 25(OH)D concentrations, and rigorous assessment of bias. The pooled sample size afforded greater power than individual trials, and risk of bias was assessed as being low in three of four studies. Contour-enhanced funnel plots and Egger's test (P = 0.71) indicated no publication bias, contrasting with earlier observational meta-analyses reporting significant small-study effects.^8^ This methodological rigour strengthens confidence in the validity of our null findings.

Limitations include: heterogeneity in ARI case definitions—ranging from parent-reported symptoms to laboratory confirmation—may have introduced misclassification bias, potentially diluting observed effects. Reliance on aggregate rather than individual participant data (IPD). This constrains adjustments for covariates and precise subgroup analyses. The small number of trials limited power to detect modest effects, particularly in subgroups with baseline 25(OH)D < 25 nmol/L where few participants were enrolled, and limited our subgroup analysis and subsequent meta-regression of potential effect modifiers, however we sought outcome data stratified by Maternal baseline vitamin D status to partially address this limitation. Furthermore, most trials were conducted in settings where standard care included low-dose vitamin D, limiting generalisability.

Future research should prioritise IPD meta-analyses to explore individual-level effect modifiers, including investigations of cord blood 25(OH)D levels and their relationship to offspring ARI incidence. RCTs targeting populations with high prevalence of profound vitamin D deficiency, using sufficient doses are needed to provide definitive clarification of whether benefits may exist in high-risk subgroups. However, ethical constraints relating to withholding vitamin D supplementation from deficient mothers randomised to the control arm of such trials should be acknowledged. Additionally, mechanistic studies evaluating the impact of gestational vitamin D on offspring immune trajectories, including responses to vaccines, could elucidate pathways not captured by ARI incidence alone.

In conclusion, this meta-analysis provides high-quality evidence that maternal vitamin D supplementation during pregnancy does not reduce offspring ARI risk, regardless of baseline maternal vitamin D status. These findings do not support routine antenatal vitamin D supplementation for ARI prevention in offspring. However, targeted supplementation in high-risk, deficient populations may warrant further investigation.

## Contributors

DAJ and ARM wrote the study protocol and designed statistical analyses; assessed eligibility of studies for inclusion and performed risk of bias assessments. DAJ and ARM had access to, and verified, the underlying data from all original research articles. Statistical analyses were done by DAJ; results were checked and verified by JDS. DAJ and ARM wrote the first draft of the report. All authors revised it critically for important intellectual content, gave final approval of the version to be published, and agreed to be accountable for all aspects of the work in ensuring that questions related to the accuracy or integrity of any part of the work were appropriately investigated and resolved.

## Data sharing statement

The study dataset is available with publication by reasonable request made to d.a.jolliffe@qmul.ac.uk.

## Declaration of interests

All authors have completed the ICMJE uniform disclosure form. **STW** receives royalties from UpToDate and is on the board of Histolix, a digital pathology company. **SKM** has received honoraria from Pfizer, GlaxoSmithKline, and Sanofi Pasteur for lectures and ad hoc advisory boards, all unrelated to this study. **NCH** reports fees, consultancy, lecture fees and/or honoraria from AMGEN, UCB, Echolight, Kyowa Kirin, Theramex outside the submitted work. **AAL** reports grants from US National Institutes of Health research, royalties from UpToDate and is an advisory board member of US National Institutes of Health, National Heart, Lung, and Blood Institute. **ARM**, **DAJ, CC, NB, BC, SD, RM** and **JDS** declare no competing interests. The views expressed in this publication are those of the author(s).

## Appendix A Supplementary data

Supplemntary Figures and Tables
