# Corrigendum to Association between maternal vitamin D supplementation during pregnancy and the risk of acute respiratory infections in offspring: a systematic review and meta-analysis

**DOI:** 10.1016/j.eclinm.2026.103762

**Published:** 2026-01-21

**Authors:** David A. Jolliffe, Nicklas Brustad, Bo Chawes, Cyrus Cooper, Stefania D'angelo, Nicholas C. Harvey, Augusto A. Litonjua, Rebecca Moon, Shaun K. Morris, John D. Sluyter, Scott T. Weiss, Adrian R. Martineau

**Affiliations:** a Blizard Institute, Faculty of Medicine and Dentistry, Queen Mary University of London, London, UK; b COPSAC, Copenhagen Prospective Studies on Asthma in Childhood, Herlev and Gentofte Hospital, University of Copenhagen, Copenhagen, Denmark; c MRC Lifecourse Epidemiology Unit, University of Southampton, Southampton, UK; d Department of Pediatrics Golisano Children's Hospital, Pediatric Pulmonary Division, University of Rochester Medical School, Rochester, NY, USA; e Division of Infectious Diseases, Child Health Evaluative Sciences & Centre for Global Child Health, Hospital for Sick Children, 686 Bay Street, Toronto, ON, Canada; f School of Population Health, University of Auckland, Auckland, New Zealand; g Department of Medicine, Channing Division of Network Medicine, Brigham and Women's Hospital, Harvard Medical School, Boston, MA, USA; h NIHR Southampton Biomedical Research Centre, University of Southampton and University Hospital NHS Foundation Trust, Southampton, UK

Corrigendum: Barts Charity funding of DAJ's salary was omitted in error. The first paragraph of the Acknowledgments section is corrected to “*This study was conducted without external funding. ARM is supported by the* *United Kingdom Office for Students*. *DAJ is supported by* 10.13039/100015652*Barts Charity* *(G-002700). The views expressed are those of the authors and not necessarily those of Barts Charity or the Office for Students. Sources of support for individual trials are detailed in Appendix p7. We thank all the people who participated in primary randomised controlled trials, and the teams who conducted them.”* The Role of the funding source statement is also corrected to *“The funder of the study had no role in study design, data collection, data analysis, data interpretation, or writing of the report”.*

