# Supplementary material for: Association between maternal vitamin D supplementation during pregnancy and the risk of acute respiratory infections in offspring: a systematic review and meta-analysis
Source: eClinicalMedicine. 2025 Dec 4;90:103682. doi: 10.1016/j.eclinm.2025.103682 (PMC12720986; doi:10.1016/j.eclinm.2025.103682)
Supplement: Supplemntary Figures and Tables [file mmc1.pdf]

Association between maternal vitamin D supplementation during pregnancy and the risk of acute respiratory infections in offspring: a systematic review and meta-analysis

Appendix

Table of Contents

1. **Search strategies** 3
2. **Data Collection Processes** 6
3. **Sources of support for participating trials** 7
4. **Registration and Ethical Approval for primary trials contributing data to this meta-analysis** 8
5. **Appendix Table 1: Reasons for exclusion of potentially relevant studies** 9
6. **Appendix Table 2: Risk of Bias Assessment** 10
7. **Appendix Table 3: Summary of Findings Table** 11
8. **Appendix Table 4: Results of Sensitivity and Exploratory analyses** 12
9. **Appendix Figure S2: Forest plot of RCTs comparing effects of vitamin D vs. placebo, reporting rate of acute respiratory infections in offspring, by maternal baseline 25-hydroxyvitamin D level. A) <25.0 nmol/L; B) 25.0 to 49.9 nmol/L; C) 50.0 to 74.9 nmol/L, and D) ≥75.0 nmol/L.** 13
10. **Appendix Figure S3: Forest plot of RCTs comparing effects of maternal supplementation with vitamin D vs. placebo on incidence of upper respiratory infections in offspring** 14
11. **Appendix Figure 4: Forest plot of RCTs comparing effects of maternal supplementation with vitamin D vs. placebo on incidence of lower respiratory infections in offspring** 15
12. **Appendix Figure S5: Funnel plot of placebo-controlled RCTs reporting incidence of ARI in offspring, overall (primary comparison)** 16
13. **Appendix Figure S6: Leave-one-out Sensitivity Analysis: Forests plots of RCTs comparing effects of maternal supplementation with vitamin D vs. placebo on incidence of ARI in offspring.** 17

1. Search strategies

A. Medline

Cochrane Highly Sensitive Search Strategy for identifying randomised controlled trials

#1. randomized controlled trial [pt] OR controlled clinical trial [pt] OR randomized [tiab] OR placebo [tiab] OR drug therapy [sh] OR randomly [tiab] OR trial [tiab] OR groups [tiab]

#2. animals [mh] NOT humans [mh]

#3. #1 NOT #2

Terms specific to vitamin D

#4. Vitamin D OR vitamin D2 OR vitamin D3 OR cholecalciferol OR ergocalciferol OR alphacalcidol OR alfacalcidol OR calcitriol OR paricalcitol OR doxerocalciferol

Terms specific to acute respiratory infection

#5. Acute Respiratory Infection OR Upper Respiratory Infection OR Lower Respiratory Infection OR Respiratory Tract Infection OR Common Cold OR Sinusitis OR Pharyngitis OR Laryngitis OR Laryngotracheobronchitis OR Tonsillitis OR peritonsillar abscess OR Croup OR Epiglottitis OR supraglottitis OR Otitis Media OR Pneumonia OR Bronchopneumonia OR Bronchitis OR Bronchiolitis OR Pleurisy OR Pleuritis OR Wheez* OR Respiratory OR Asthma

Terms specific to study population

#6. Antenatal [tiab] OR Prenatal [Tiab] OR Maternal [Tiab] OR Mother [Tiab] OR Pregnan* [Tiab]

Combination of terms to identify randomised controlled trials of maternal vitamin D supplementation during pregnancy for the prevention of acute respiratory infections in offspring.

#3 AND #4 AND #5 AND #6

B. EMBASE

Terms for identifying randomised controlled trials

#1. ‘randomized controlled trial’/exp OR ‘single blind procedure’/exp OR ‘double blind procedure’/exp OR ’crossover procedure’/exp

#2. random*:ab,ti OR placebo*:ab,ti OR crossover*:ab,ti OR ’cross over’:ab,ti OR allocat*:ab,ti OR ((singl* OR doubl*) NEXT/1 blind*):ab,ti OR trial:ti

#3. #1 OR #2

Terms specific to vitamin D

#4. *vitamin AND d OR vitamin AND d2 OR vitamin AND d3 OR cholecalciferol OR ergocalciferol OR alphacalcidol OR alfacalcidol OR calcitriol OR paricalcitol OR doxerocalciferol*

Terms specific to acute respiratory infection

#5. acute AND respiratory AND infection OR upper AND respiratory AND infection OR lower AND respiratory AND infection OR respiratory AND tract AND infection OR common AND cold OR sinusitis OR pharyngitis OR laryngitis OR laryngotracheobronchitis OR tonsillitis OR peritonsillar AND abscess OR croup OR epiglottitis OR supraglottitis OR otitis AND media OR pneumonia OR bronchopneumonia OR bronchitis OR bronchiolitis OR pleurisy OR pleuritis OR wheez* OR respiratory OR asthma

Terms specific to study population

#6. antenatal OR arenatal OR maternal OR mother OR pregnan*

Combination of terms to identify randomised controlled trials of maternal vitamin D supplementation during pregnancy for the prevention of acute respiratory infections in offspring.

#3 AND #4 AND #5 AND #6

C. Cochrane Central

Terms specific to vitamin D

#1. Vitamin D OR vitamin D2 OR vitamin D3 OR cholecalciferol OR ergocalciferol OR alphacalcidol OR alfacalcidol OR calcitriol OR paricalcitol OR doxerocalciferol

Terms specific to acute respiratory infection

#2. Acute Respiratory Infection OR Upper Respiratory Infection OR Lower Respiratory Infection OR Respiratory Tract Infection OR Common Cold OR Sinusitis OR Pharyngitis

OR Laryngitis OR Laryngotracheobronchitis OR Tonsillitis OR peritonsillar abscess OR Croup OR Epiglottitis OR supraglottitis OR Otitis Media OR Pneumonia OR Bronchopneumonia OR Bronchitis OR Bronchiolitis OR Pleurisy OR Pleuritis OR Wheez* OR Respiratory OR Asthma

Terms specific to study population

#3. Antenatal OR Prenatal OR Maternal OR Mother OR Pregnan*

Combination of terms to identify randomised controlled trials of maternal vitamin D supplementation during pregnancy for the prevention of acute respiratory infections in offspring.

#1 AND #2 AND #3

D. Web of Science

TS =(Vitamin D OR vitamin D2 OR vitamin D3 OR cholecalciferol OR ergocalciferol OR alphacalcidol OR alfacalcidol OR calcitriol OR paricalcitol OR doxerocalciferol) AND TS =(Acute Respiratory Infection OR Upper Respiratory Infection OR Lower Respiratory Infection OR Respiratory Tract Infection OR Common Cold OR Sinusitis OR Pharyngitis OR Laryngitis OR Laryngotracheobronchitis OR Tonsillitis OR peritonsillar abscess OR Croup OR Epiglottitis OR supraglottitis OR Otitis Media OR Pneumonia OR Bronchopneumonia OR Bronchitis OR Bronchiolitis OR Pleurisy OR Pleuritis OR Wheez* OR Respiratory OR Asthma) AND TS =(placebo* or random* or clinical trial* or double blind* or single blind* or rct) AND TI =(Antenatal OR Prenatal OR Maternal OR Mother OR Pregnan*)

E. ClinicalTrials.gov

Vitamin D AND respiratory AND infection AND antenatal OR prenatal OR maternal OR mother OR pregnan*

2. Data Collection Processes

Summary data relating to the primary outcome (overall and by sub-group) and secondary outcomes (overall only) from identified trials were requested from Principal Investigators. On receipt, they were assessed for consistency with associated publications. Study authors were contacted to provide missing data and to resolve any queries arising from these consistency checks. Once queries had been resolved, clean summary data were uploaded to the study database, which was held in STATA IC v14.2 (StataCorp, College Station, TX).

Data were extracted for the following variables: study setting, eligibility criteria, maternal age, offspring sex, maternal baseline vitamin D status, details of intervention and control regimens, trial duration, case definitions for ARI, and numbers of participants contributing data to statistical analyses. Follow-up summary data were requested for the total number of ARI events and years at risk during the trial, both overall and stratified by potential effect modifiers, where this was available. We also requested summary data on the total number of the following events during the trial: upper respiratory infection (URI); lower respiratory infection (LRI); Emergency Department attendance and/or hospital admission for ARI; death due to ARI or respiratory failure; use of antibiotics to treat an ARI; absence from work or school due to ARI; a serious adverse event; death due to any cause; and potential adverse reactions to vitamin D (hypercalcaemia and renal stones).

3. Sources of support for participating trials

The trial by Cooper and colleagues was supported by grants from Arthritis Research UK, Medical Research Council (MRC), Bupa Foundation, National Institute for Health Research (NIHR) Southampton Biomedical Research Centre, University of Southampton and University Hospital Southampton NHS Foundation Trust, and NIHR Musculoskeletal Biomedical Research Unit, University of Oxford. International Standard Randomised Controlled Trial registry, ISRCTN 82927713, and the European Clinical Trials Database, EudraCT 2007–001716–23.

The trial by Chawes and colleagues was supported by private and public research funds listed on http://www.copsac.com. Clinicaltrials.gov number, NCT00856947.

The trial by Litonjua and colleagues was supported by the National Heart, Lung, and Blood Institute; VDAART. ClinicalTrials.gov number, NCT00920621.

The trial by Morris and colleagues was supported by the Bill and Melinda Gates Foundation (grant number OPP1066764). ClinicalTrials.gov number, NCT02388516.

4. Registration and Ethical Approval for primary trials contributing data to this meta-analysis

Research Ethics Committee approval to contribute aggregate data to this meta-analysis were not required in the UK, USA, Denmark or Bangladesh.

5. Appendix Table 1: Reasons for exclusion of potentially relevant studies

**First author, year or clinicaltrials.gov registration number**	**Reason for exclusion**
Goldring, 2013[1]	Ineligible: not placebo controlled
Chandy, 2016[2]	Ineligible: intervention of vitamin D3 was administered to offspring
Grant, 2016[3]	Ineligible: intervention of vitamin D3 was administered to offspring
Nasantogtokh, 2023[4]	Ineligible: intervention of vitamin D3 was administered to Mothers during and after pregnancy
NCT01229189	Ineligible: unpublished; completion status unconfirmed

6. Appendix Table 2: Risk of Bias Assessment

	Sequence generation	Allocation concealment	Blinding of participants and personnel	Blinding of outcome assessment	Incomplete outcome data	Selective reporting	Other bias
Chawes, 2016[5]	✓	✓	✓	✓	✓	✓	✓
Cooper, 2016[6]	✓	✓	✓	✓	?	✓	✓
Litonjua, 2016[7]	✓	✓	✓	✓	✓	✓	✓
Morris, 2021[8]	✓	✓	✓	✓	✓	✓	✓

✓ = low risk of bias; ? = unclear risk of bias, N/A = not applicable (unpublished)

7. Appendix Table 3: Summary of Findings Table

Vitamin D3 compared to placebo for prevention of acute respiratory infection (ARI)

Population: children and adults of any age, sex or ethnic origin, with or without co-morbidity

Setting: Eighteen countries on four continents (Asia, Australasia, Europe, North America)

Intervention: oral vitamin D_3 (cholecalciferol) supplementation

Comparison: oral placebo

Outcomes	Anticipated absolute effects* (95% CI)		Relative effect (95% CI)	№ of participants (studies)	Quality of the evidence (GRADE)
	**Risk with placebo**	**Risk with Vitamin D**			
Overall rate of incident **ARI** among offspring of mothers	The mean overall rate of was **4.52** per person-year	4.57 per person-year (4.43 to 4.70)	1.01 (0.98 to 1.03)	3084 (4 RCTs)	⊕⊕⊕ MODERATE
Rate of incident **URI** among offspring of mothers	The mean overall rate of was **4.25** per person-year	4.29 per person-year (4.16 to 4.42)	1.01 (0.98 to 1.04)	3084 (4 RCTs)	⊕⊕⊕ MODERATE
Rate of incident **LRI** among offspring of mothers	The mean overall rate of was **0.28** per person-year	0.29 per person-year (0.23 to 0.31)	0.95 (0.82 to 1.09)	3084 (4 RCTs)	⊕⊕⊕ MODERATE

***The risk in the intervention group** (and its 95% confidence interval) is based on the assumed risk in the comparison group and the **relative effect** of the intervention (and its 95% CI).

CI: Confidence interval; **IRR:** Incidence Rate Ratio

GRADE Working Group grades of evidence
High quality: We are very confident that the true effect lies close to that of the estimate of the effect
Moderate quality: We are moderately confident in the effect estimate: The true effect is likely to be close to the estimate of the effect, but there is a possibility that it is substantially different
Low quality: Our confidence in the effect estimate is limited: The true effect may be substantially different from the estimate of the effect
Very low quality: We have very little confidence in the effect estimate: The true effect is likely to be substantially different from the estimate of effect

8. Appendix Table 4: Results of Sensitivity and Exploratory analyses

	No. trials	Intervention group			Control group			Incidence Rate Ratio (95% CI)	I^2%	P for heterog-eneity
		Offspring, N	Total ARI events	Person-years	Offspring, N	Total ARI events	Person-years			
**Sensitivity analysis: excluding one study with unclear risk of bias**	3	1,401	10,442	2,468.7	916	9,968	2,172.4	0.99 (0.97 to 1.02)	0.0	0.49

9. Appendix Figure S2: Forest plot of RCTs comparing effects of vitamin D vs. placebo, reporting rate of acute respiratory infections in offspring, by maternal baseline 25-hydroxyvitamin D level. A) <25.0 nmol/L; B) 25.0 to 49.9 nmol/L; C) 50.0 to 74.9 nmol/L, and D) ≥75.0 nmol/L.

A

1st Author (trial)	Year	ARI Incidence Rate, intervention group	ARI Incidence Rate, control group	Incident Rate Ratio (95% CI)	% Weight
Cooper	2016	4.167488	3.333333	1.25 (1.09, 1.43)	34.83
Chawes	2016	5.866667	4.666667	1.26 (0.87, 1.82)	10.04
Litonjua	2016	5.421052	5.28125	1.03 (0.91, 1.15)	38.14
Morris	2021	1.267344	1.262626	1.00 (0.77, 1.30)	16.99
Overall (I-squared = 47.8%, p = 0.124)				1.12 (0.98, 1.27)	100.00

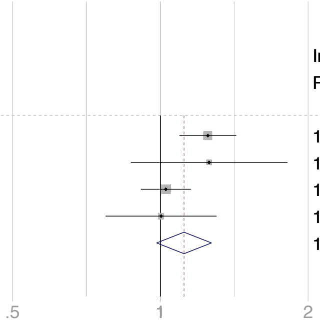

NOTE: Weights are from random effects analysis

B

1st Author (trial)	Year	ARI Incidence Rate, intervention group	ARI Incidence Rate, control group	Incident Rate Ratio (95% CI)	% Weight
Cooper	2016	4.431738	4.431292	1.00 (0.95, 1.06)	36.03
Chawes	2016	4.426357	4.611111	0.96 (0.85, 1.09)	21.76
Litonjua	2016	5.185792	4.615942	1.12 (1.05, 1.20)	34.26
Morris	2021	1.315169	1.161616	1.13 (0.86, 1.48)	7.95
Overall (I-squared = 68.5%, p = 0.023)				1.04 (0.96, 1.13)	100.00

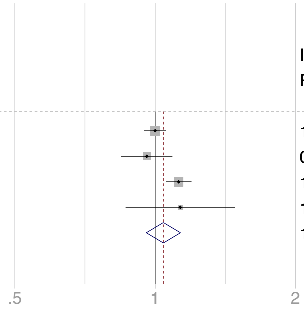

NOTE: Weights are from random effects analysis

C

1st Author (trial)	Year	ARI Incidence Rate, intervention group	ARI Incidence Rate, control group	Incident Rate Ratio (95% CI)	% Weight
Cooper	2016	4.789574	4.640805	1.03 (0.96, 1.10)	33.14
Chawes	2016	5.366301	5.112211	1.05 (0.98, 1.13)	32.12
Litonjua	2016	4.002267	4.326577	0.93 (0.87, 0.99)	33.89
Morris	2021	1.515152	1.178451	1.29 (0.57, 2.89)	0.86
Overall (I-squared = 64.9%, p = 0.036)				1.00 (0.93, 1.08)	100.00

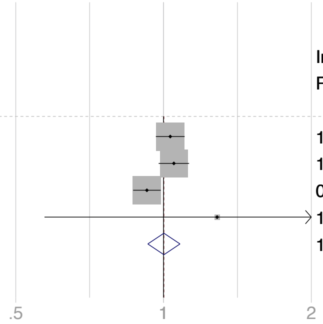

NOTE: Weights are from random effects analysis

D

1st Author (trial)	Year	ARI Incidence Rate, intervention group	ARI Incidence Rate, control group	Incident Rate Ratio (95% CI)	% Weight
Cooper	2016	5.145889	4.217506	1.22 (0.99, 1.50)	12.88
Chawes	2016	5.062222	5.365297	0.94 (0.89, 1.00)	47.86
Litonjua	2016	4.238596	4.539094	0.93 (0.86, 1.01)	39.12
Morris	2021	1.111111	.9259259	1.20 (0.12, 11.54)	0.14
Overall (I-squared = 47.6%, p = 0.126)				0.97 (0.89, 1.06)	100.00

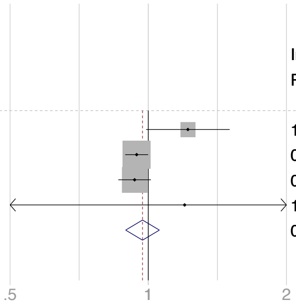

NOTE: Weights are from random effects analysis

10. Appendix Figure S3: Forest plot of RCTs comparing effects of maternal supplementation with vitamin D vs. placebo on incidence of upper respiratory infections in offspring

1st Author (trial)	Year	ARI Incidence Rate, intervention group	ARI Incidence Rate, control group	Incident Rate Ratio (95% CI)	% Weight
Cooper	2016	4.112523	3.967745	1.04 (0.99, 1.08)	31.82
Chawes	2016	4.862543	4.991696	0.97 (0.93, 1.02)	30.72
Litonjua	2016	4.369547	4.304239	1.02 (0.98, 1.05)	34.64
Morris	2021	1.292356	1.21874	1.06 (0.88, 1.27)	2.81
Overall (I-squared = 35.0%, p = 0.202)				1.01 (0.98, 1.04)	100.00

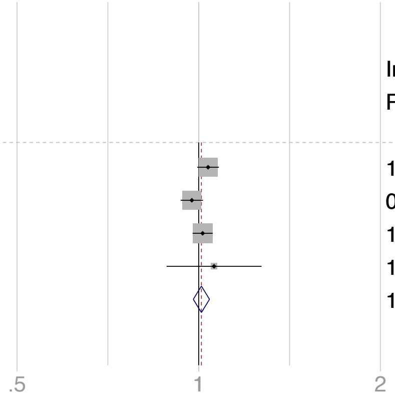

NOTE: Weights are from random effects analysis

11. Appendix Figure 4: Forest plot of RCTs comparing effects of maternal supplementation with vitamin D vs. placebo on incidence of lower respiratory infections in offspring

1st Author (trial)	Year	ARI Incidence Rate, intervention group	ARI Incidence Rate, control group	Incident Rate Ratio (95% CI)	% Weight
Cooper	2016	.3938294	.4080905	0.97 (0.85, 1.10)	37.85
Chawes	2016	.1958763	.1803084	1.09 (0.87, 1.35)	24.24
Litonjua	2016	.1827161	.2294264	0.80 (0.67, 0.95)	30.10
Morris	2021	.1943788	.1741057	1.12 (0.69, 1.80)	7.80
Overall (I-squared = 47.6%, p = 0.126)				0.95 (0.82, 1.09)	100.00

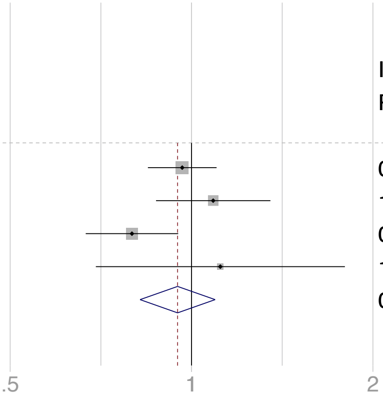

NOTE: Weights are from random effects analysis

12. Appendix Figure S5: Funnel plot of placebo-controlled RCTs reporting incidence of ARI in offspring, overall (primary comparison)

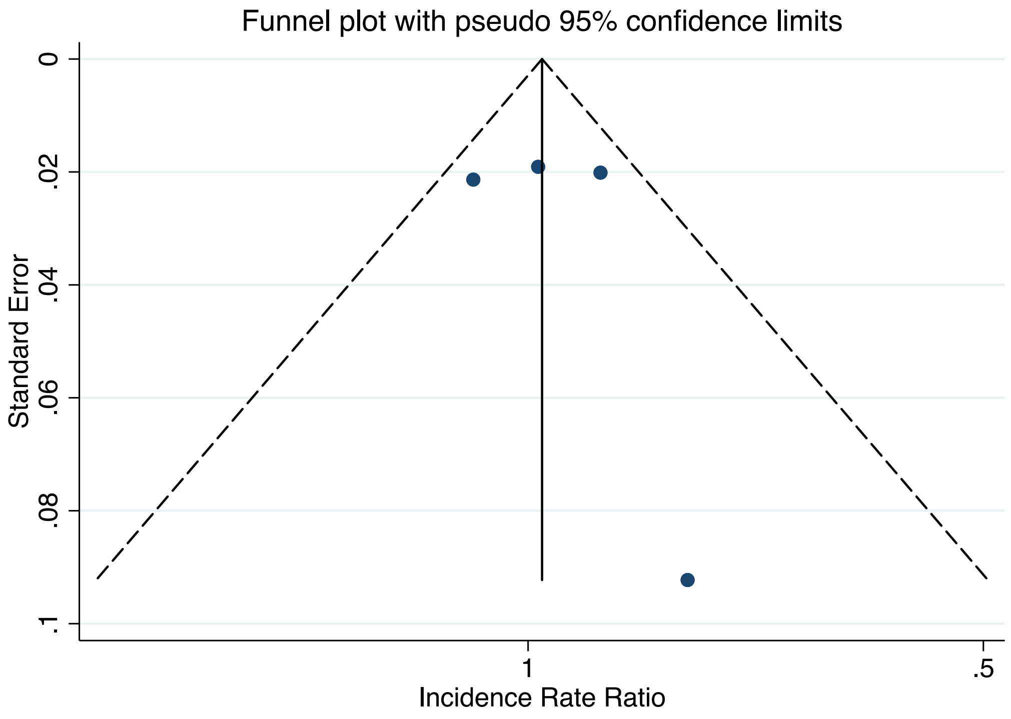

13. Appendix Figure S6: Leave-one-out Sensitivity Analysis: Forests plots of RCTs comparing effects of maternal supplementation with vitamin D vs. placebo on incidence of ARI in offspring.

Excluding Cooper et al.

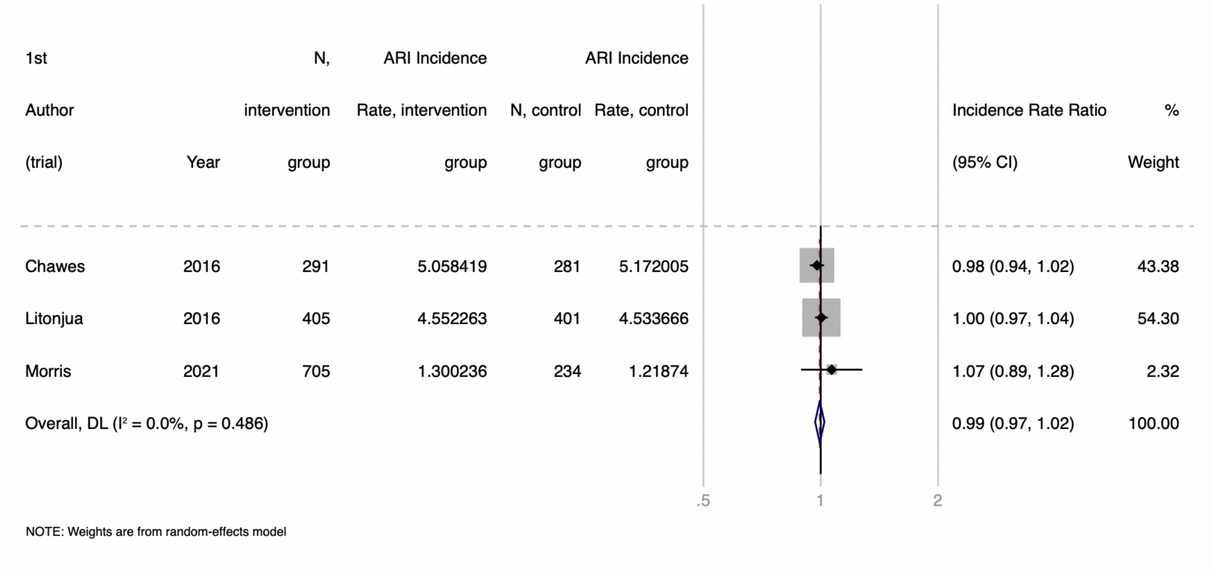

Excluding Chawes et al.

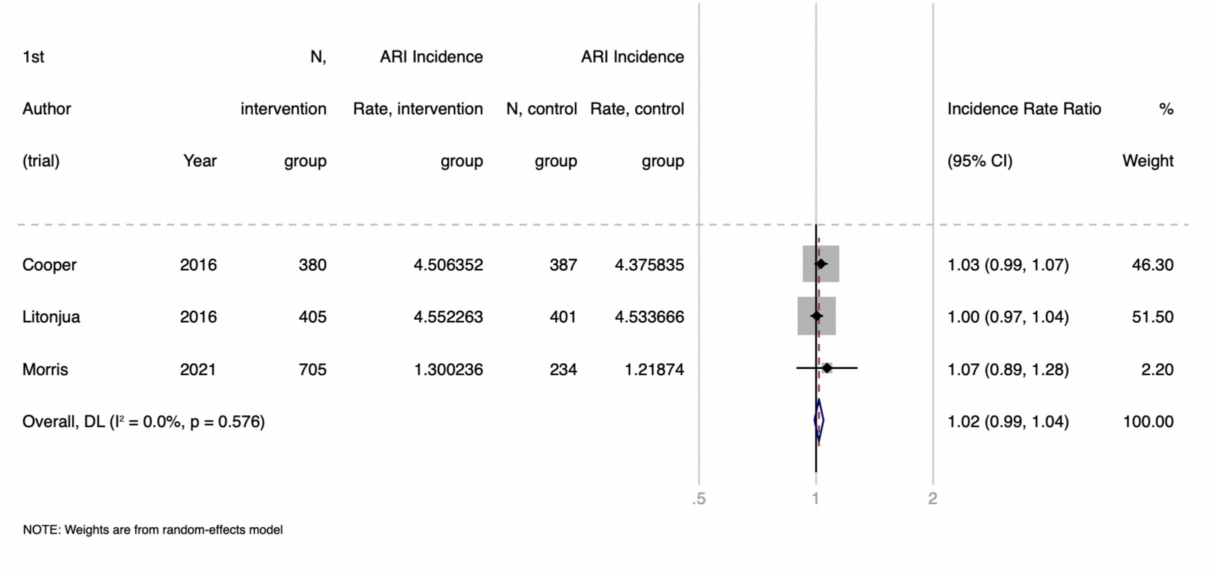

Excluding Litonjua et al.

1st Author (trial)	Year	N, intervention group	ARI Incidence Rate, intervention group	N, control group	ARI Incidence Rate, control group	Incidence Rate Ratio (95% CI)	% Weight
Cooper	2016	380	4.506352	387	4.375835	1.03 (0.99, 1.07)	48.49
Chawes	2016	291	5.058419	281	5.172005	0.98 (0.94, 1.02)	46.16
Morris	2021	705	1.300236	234	1.21874	1.07 (0.89, 1.28)	5.35
Overall, DL (I^2 = 42.9%, p = 0.174)						1.01 (0.96, 1.05)	100.00

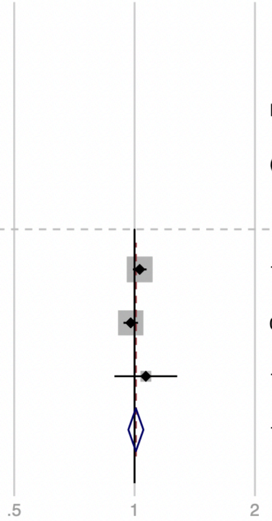

NOTE: Weights are from random-effects model

Excluding Morris et al.

1st Author (trial)	Year	N, intervention group	ARI Incidence Rate, intervention group	N, control group	ARI Incidence Rate, control group	Incidence Rate Ratio (95% CI)	% Weight
Cooper	2016	380	4.506352	387	4.375835	1.03 (0.99, 1.07)	33.39
Chawes	2016	291	5.058419	281	5.172005	0.98 (0.94, 1.02)	30.89
Litonjua	2016	405	4.552263	401	4.533666	1.00 (0.97, 1.04)	35.72
Overall, DL (I^2 = 35.4%, p = 0.213)						1.00 (0.98, 1.03)	100.00

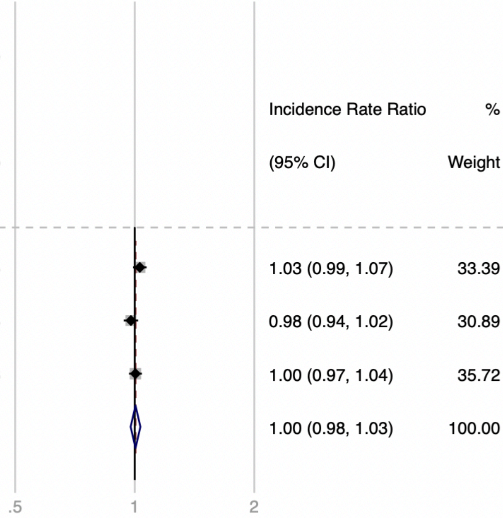

NOTE: Weights are from random-effects model

References

1. Goldring ST, Griffiths CJ, Martineau AR, et al. Prenatal vitamin d supplementation and child respiratory health: a randomised controlled trial. *PLoS One* 2013; **8**(6): e66627.
2. Chandy DD, Kare J, Singh SN, et al. Effect of vitamin D supplementation, directly or via breast milk for term infants, on serum 25 hydroxyvitamin D and related biochemistry, and propensity to infection: a randomised placebo-controlled trial. *Br J Nutr* 2016; **116**(1): 52-8.
3. Grant CC, Crane J, Mitchell EA, et al. Vitamin D supplementation during pregnancy and infancy reduces aeroallergen sensitization: a randomized controlled trial. *Allergy* 2016; **71**(9): 1325-34.
4. Nasantogtokh E, Ganmaa D, Altantuya S, Amgalan B, Enkhmaa D. Maternal vitamin D intakes during pregnancy and child health outcome. *J Steroid Biochem Mol Biol* 2023; **235**: 106411.
5. Chawes BL, Bonnelykke K, Stokholm J, et al. Effect of Vitamin D3 Supplementation During Pregnancy on Risk of Persistent Wheeze in the Offspring: A Randomized Clinical Trial. *JAMA* 2016; **315**(4): 353-61.
6. Cooper C, Harvey NC, Bishop NJ, et al. Maternal gestational vitamin D supplementation and offspring bone health (MAVIDOS): a multicentre, double-blind, randomised placebo-controlled trial. *Lancet Diabetes Endocrinol* 2016; **4**(5): 393-402.
7. Litonjua AA, Carey VJ, Laranjo N, et al. Effect of Prenatal Supplementation With Vitamin D on Asthma or Recurrent Wheezing in Offspring by Age 3 Years: The VDAART Randomized Clinical Trial. *JAMA* 2016; **315**(4): 362-70.
8. Morris SK, Pell LG, Rahman MZ, et al. Effects of Maternal Vitamin D Supplementation During Pregnancy and Lactation on Infant Acute Respiratory Infections: Follow-up of a Randomized Trial in Bangladesh. *J Pediatric Infect Dis Soc* 2021; **10**(9): 901-9.
